# The use of healthcare simulation to identify and address latent safety threats: a scoping review

**DOI:** 10.3389/frhs.2025.1682629

**Published:** 2025-11-18

**Authors:** Olivia Lounsbury, Ashley Tomlinson, Judy Wakeling, Paul Bowie, Helen Higham

**Affiliations:** 1 Nuffield Department of Surgical Sciences, University of Oxford, Oxford, United Kingdom; 2 Oxford Simulation, Teaching, and Research Centre, Oxford, United Kingdom; 3 NHS Education for Scotland, Glasgow, United Kingdom; 4 Centre for Health Innovation, University of Staffordshire, Stafford, United Kingdom

**Keywords:** healthcare simulation, patient safety, safety risks, system engineering, latent safety threats, quality improvement

## Abstract

**Background:**

Simulation is a well-established tool for clinical education and has been used to uncover latent safety threats (LSTs) in healthcare settings. However, the extent to which systems theory underpins efforts to detect and mitigate LSTs remains unclear.

**Objective:**

This scoping review explores how healthcare simulations have been used to identify and address LSTs, with particular attention to the visibility and application of systems theory in study design, implementation, and analysis.

**Methods:**

Using PRISMA-ScR, we systematically reviewed studies from 2014 to 2024 across MEDLINE, EMBASE, and grey literature sources. Studies were included if simulation was used with the primary aim of identifying LSTs. Data extraction focused on definitions of LSTs, approaches used to identify and analyse LSTs, response strategies, and the visibility of systems theory.

**Results:**

Sixty-six studies met inclusion criteria. Most (74.2%) used the term “latent safety threat,” though definitions varied. Many studies lacked explicit detail on how LSTs were identified (33.3%) or analysed (41.8%). Systems theory was applied with varying visibility: 36.4% showed unclear or no visibility, 43.9% showed partial visibility, and 19.7% showed full visibility. While 80.3% described actions to address LSTs, approaches ranged from one-off fixes to structured quality improvement strategies. Case studies illustrate best practices and opportunities for improvement in theoretical transparency.

**Conclusions:**

Simulation is a valuable method for identifying LSTs, but inconsistent application of systems theory and variable methodological transparency limit learning and generalisability. Future research should make theoretical underpinnings explicit, define terminology clearly, and align simulation design with both educational and organisational improvement goals.

## Introduction

Identifying safety issues only after patient or staff harm is not uncommon. Traditional approaches to safety improvement, such as root cause analyses, often start with the harm. While these investigations can be helpful, there are often indicators of issues in day-to-day work which, if mitigated, could prevent harm from occurring in the first place (1). These issues are labelled as “latent safety threats” (LST), or “previously unrecognised system-based conditions that under certain circumstances manifest and threaten patient safety” (2). Simulation has been widely used to identify LSTs in various settings, such as emergency (3), paediatrics (4) and obstetrics (5). Studies that have used simulation to identify LSTs report a myriad of LSTs found and addressed, suggesting that the method can facilitate significant strides towards safer care. However, the approaches used to identify LSTs, and the influence of each on the nature of LSTs uncovered, remains unclear.

A recent review published by Grace and O’Malley was an important first step in better understanding how simulation has been used to identify LSTs in emergency settings (6). Authors highlighted wide-ranging methods used, detail provided, and the number and nature of LSTs identified. The study also revealed many studies were quality improvement (QI) initiatives, which aims to use existing knowledge to improve local systems, rather than research, which aims to produce new knowledge. These findings suggest that the degree to which the theory informs and is visible in simulation research is variable and impacts the LSTs identified.

In all qualitative research, the choice of method, data collection, sense making, and interpretation are influenced by the researcher's subjectivity and can impact the results (7). Theoretical visibility ensures the researcher's assumptions and rationale are made explicit and can determine the extent to which the findings are transferable. Because resources are limited in nearly all settings, there is a clear incentive to ensure that opportunities for safety learning yield the greatest insight. The use of systems theory has been shown to enhance clinical systems and therefore improve patient outcomes. In this paper, “systems theory” was defined as a way of understanding work that recognises how multiple elements interact to impact processes and outcomes, characterised by interactions between system factors, emergence, and feedback loops (8, 9). Thus far, the extent to which system theory has been used in the design of simulations to identify LSTs is unknown but could significantly impact the insights generated.

The 2023 Grace and O'Malley review was a significant step toward better understanding the potential for simulation to uncover LSTs. We intend to build on their review by examining the degree of theoretical visibility in the design, implementation and evaluation of simulations used to identify LSTs in all clinical areas. Additionally, we aim to understand the extent to which systems theory informed how the LSTs were addressed.

The key question for this work was “How has systems theory been used to identify, analyse, and address LSTs in healthcare simulations?”. The aims included:

1. Explore approaches previously used to identify and analyse LSTs in healthcare simulations.
2. Describe the extent of systems theory visibility in previous work.
3. Understand how uncovered LSTs have been addressed.

## Methods

This scoping review was conducted in five stages using the guidance from Levac and colleagues (2010) and the PRISMA Extension for Scoping Reviews (PRISMA-ScR) Checklist (10, 11). The research question was developed by safety experts, which guided the selection of the relevant studies. The study selection process was iterative and involved refining the search strategy and selection criteria with the research team. The extraction template was developed with the full team in alignment with the aims and was iterated based on discussion. Results were reported according to the overarching question and interpreted in comparison with previous literature.

### Protocol and registration

This scoping review protocol has not been registered on PROSPERO, as scoping review protocols were not accepted by PROSPERO at the time of this review.

### Eligibility criteria

See eligibility criteria below (Table 1).

**Table 1:** Eligibility criteria.

Domain	Inclusion criteria	Exclusion criteria
Language	In English or translated to English	—
Setting	All healthcare settings including, but not limited to, hospitals, mental health facilities, primary care settings, or clinical research environments	—
Date Range	2014–2024	—
Article type	Peer-reviewed original research articles or reviews; Key governmental or organisational reports	Viewpoints; Letters to the Editor; Theses; Conference presentations; Protocol papers; Theoretical papers; Books
Aim of the Articles	A primary aim of the article was to explore the use of simulation to identify LSTs	LSTs were found incidentally Simulation was primarily used for other purposes (e.g., training)

### Information sources and search strategy

EMBASE and MEDLINE were used to identify relevant publications that have applied simulation to identify LSTs in any healthcare context. Search terms used were based on previous literature and discussion with content experts. Search terms relating to this concept were iteratively developed in partnership with a librarian. Medical Subject Headings and free-text terms were searched. The librarian provided guidance on truncation and modification of the search terms as appropriate. See Supplementary Material 1: Searches.

Grey literature sources were identified based on librarian recommendations and discussions among the author group. Grey literature search strategies included backward citation tracking and searching websites of relevant organisations and journals.

### Data management and initial screening

Search results were stored in EndNote reference manager. After deduplication, results were moved into Rayyan™, a software for abstract screening and initial and full text review. 10% of articles were screened by both blinded reviewers independently and then discussed and reconciled. Thereafter, inclusion and exclusion criteria were further specified and remaining titles and abstracts were screened independently by both blinded researchers. Disagreements were reconciled between reviewers.

### Full text screening and data extraction

Articles from the initial screening were moved into Excel for full text review. Each article was screened by both reviewers independently. Authors then conducted an inductive analysis of all articles in the first review. Findings and disagreements were discussed among the research team. Based on the findings from the inductive analysis, an extraction template was co-designed by two independent researchers. All articles were re-reviewed using the extraction template. Core details were extracted from all included articles, per the extraction template below.

### Extraction template

See extraction template below (Table 2).

**Table 2:** Extraction template.

Field
Study aims
Approaches to Identifying LSTs (*Frameworks used to detect LSTs during the simulation)*
Approaches to Analysing LSTs *(Frameworks used to analyse LSTs identified during the simulation)*
Approaches to Risk Scoring LSTs, if applicable *(Frameworks used to assign a risk/priority score to the LSTs identified during the simulation)*
How LSTs Identified were Addressed *(Strategies such as organisational learning or communication plans)*
Description of How Theory Was Applied in the Introduction *(Text such as but not limited to*
Terms related to systems theory, References to models or theories that explain how accidents occur or how to analyse failures, Gaps highlighted in previous literature)
Description of How Theory Was Applied in the Methods *(Text such as but not limited to*
How systems theory informed design, sampling, data collection, or analysis Decisions made based on theory)
Description of How Theory Was Applied in the Discussion *(Text such as but not limited to*
Interpretation of results using existing systems models Discussion of how findings relate to or align with existing systems theory)

### Data synthesis

Data were analysed qualitatively and quantitatively. Quantitative synthesis consisted of categorical representation of the terms used to describe LSTs. Approaches to identify, analyse, and risk score LSTs were extracted and analysed quantitatively. The extent of theoretical visibility was determined inductively and was informed by previous work on theoretical visibility in qualitative research more broadly (7, 12). Case studies were used to illustrate examples of strong visibility of systems theory and opportunities for improvement.

### Ethical approval

Ethical approval is not required for this study, as primary data collection is not being conducted.

## Results

### Flow diagram of articles returned

**Figure 1:**
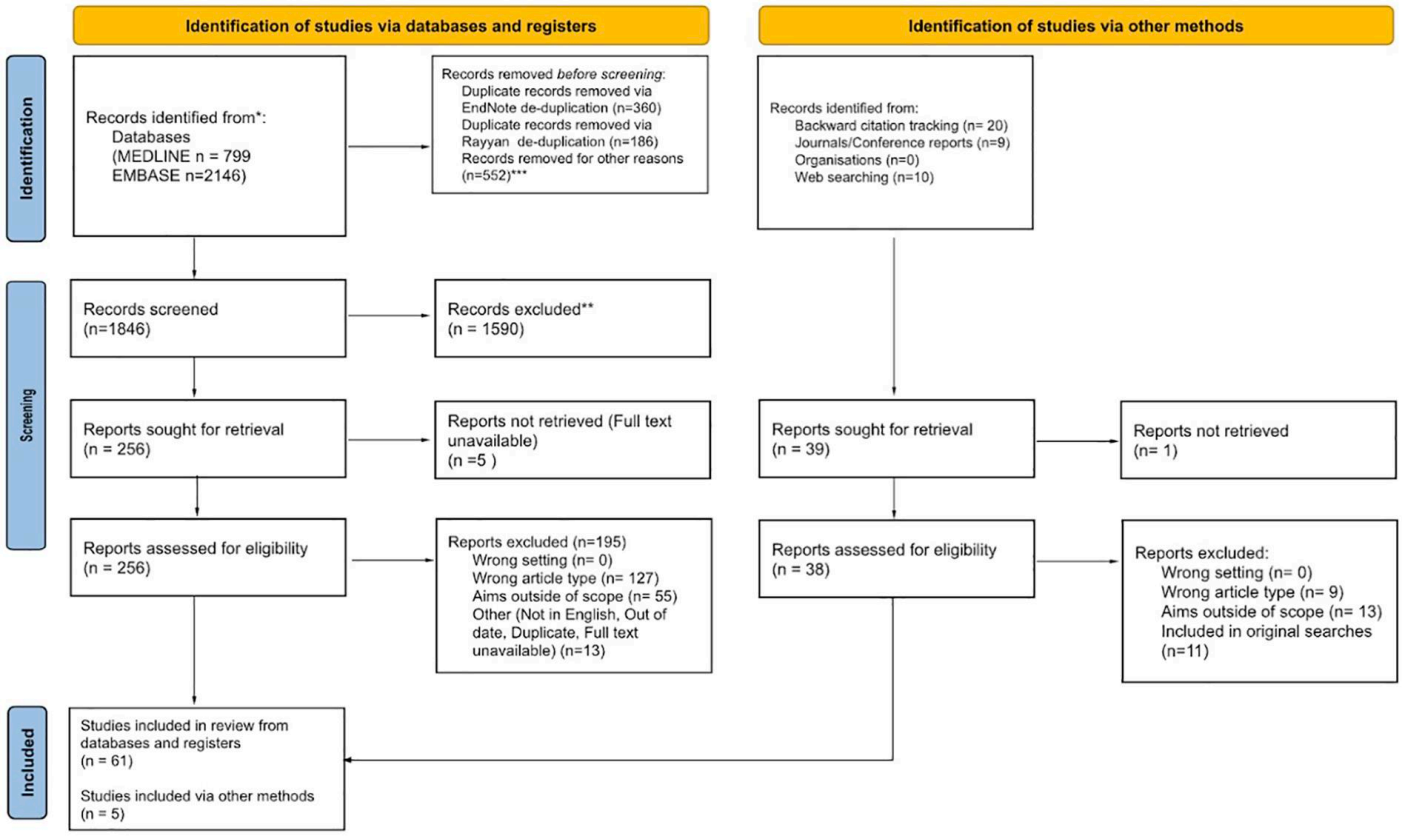
Flow diagram of articles returned.

See Figure 1 for flow diagram of articles returned.

#### Synthesis of results

##### Terminology

74.2% (49/66) articles used “Latent Safety Threat” when describing unrecognised risks the simulation aimed to uncover. Other terms included “Latent Threat” (4/66, 6.1%), “Hazard” (3/66, 4.5%), “Latent Error” (2/66, 3.0%), “Error” (2/66, 3.0%), “Failure Mode” (2/66, 3.0%), and “Safety Issues” (1/66, 1.5%). The term used was unclear in 1.5% of articles.

##### Definitions used for “latent safety threat”

63% (31/49) papers that used “latent safety threat” explicitly provided a definition. Many used the definition provided by Patterson and colleagues (2): “system-based threats to patient safety that can materialise at any time and are previously unrecognised by healthcare providers, unit directors, or hospital administration” (13–21). Nine authors specified LSTs as problems in the system (3, 22–24) or design (25–30) that could contribute to harm. Eleven authors characterised LSTs by preventability (31, 32), their unintentional nature (33, 34), significance (34, 35), or visibility using terms like “hidden” or “dormant” (31, 32, 35, 36). Kaba and Barnes (2019) and Bloomfield and colleagues (2020) described LSTs with temporal language such as “..once [a new] facility opens” (34) or “..not identified during routine patient care” (5). Broader definitions, such as “conditions that may risk patient safety” (37) and “unrecognised safety issues that impact patient outcomes” (38, 39) were also used. Arul and colleagues (2021) described LSTs not as risks but rather “improvement goals identified during simulation that have an impact on delivery of optimal care” (40).

##### Approaches used to identify LSTs

**Figure 2:**
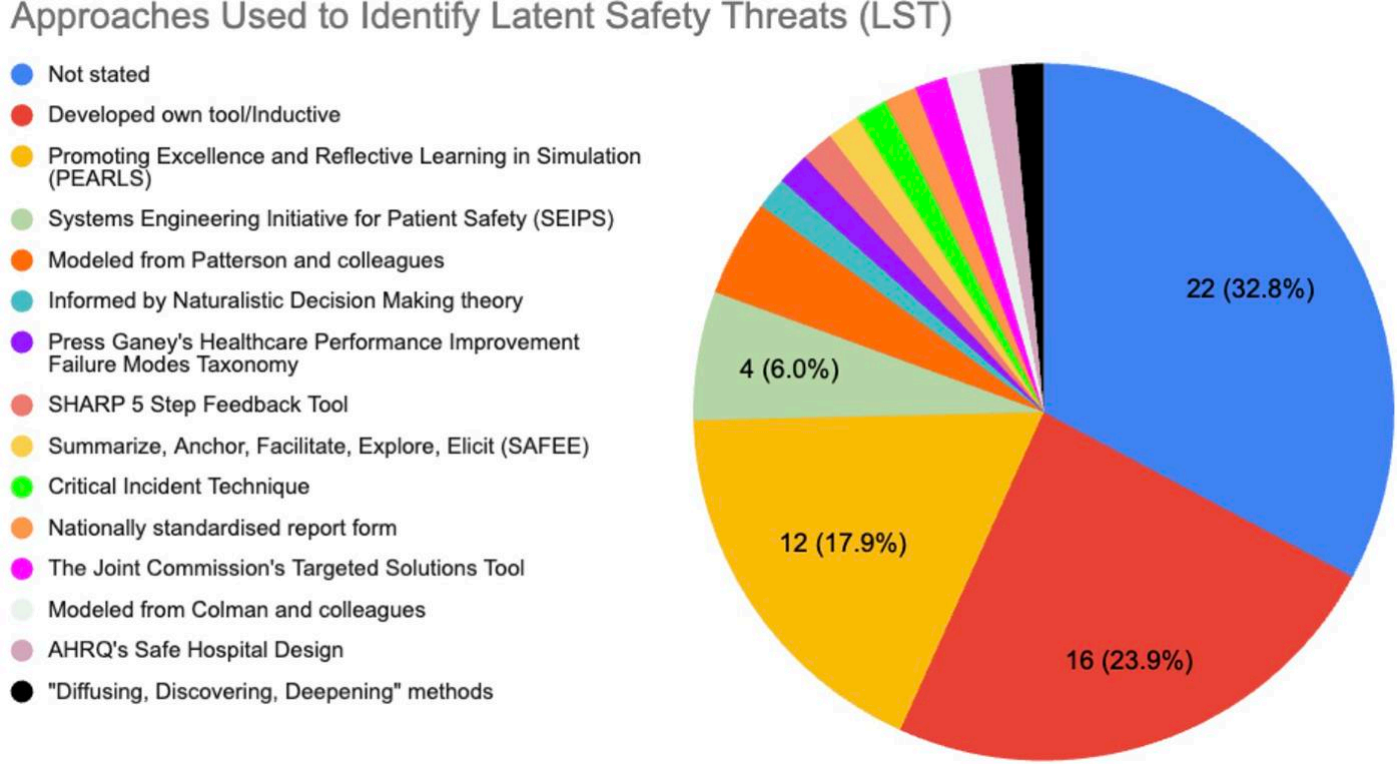
Approaches used to identify latent safety threats (LSTs). Most (32.8%, 22/66) did not state the approaches used to identify LSTs. Several (23.9%, 16/66) created their own approach. Various other tools and frameworks were used, including the Promoting Excellence and Reflective Learning in Simulation (PEARLS) and Systems Engineering Initiative for Patient Safety (SEIPS).

Several approaches were used to identify LSTs, with some authors using more than one approach. 33.3% (22/66) did not state the approach(es) used to identify LSTs. 24.2% (16/66) authors developed their own approach inductively and 18.2% (12/66) followed the Promoting Excellence and Reflective Learning in Simulation (PEARLS) framework (41). See Figure 2.

##### Approaches used to analyse LSTs

**Figure 3:**
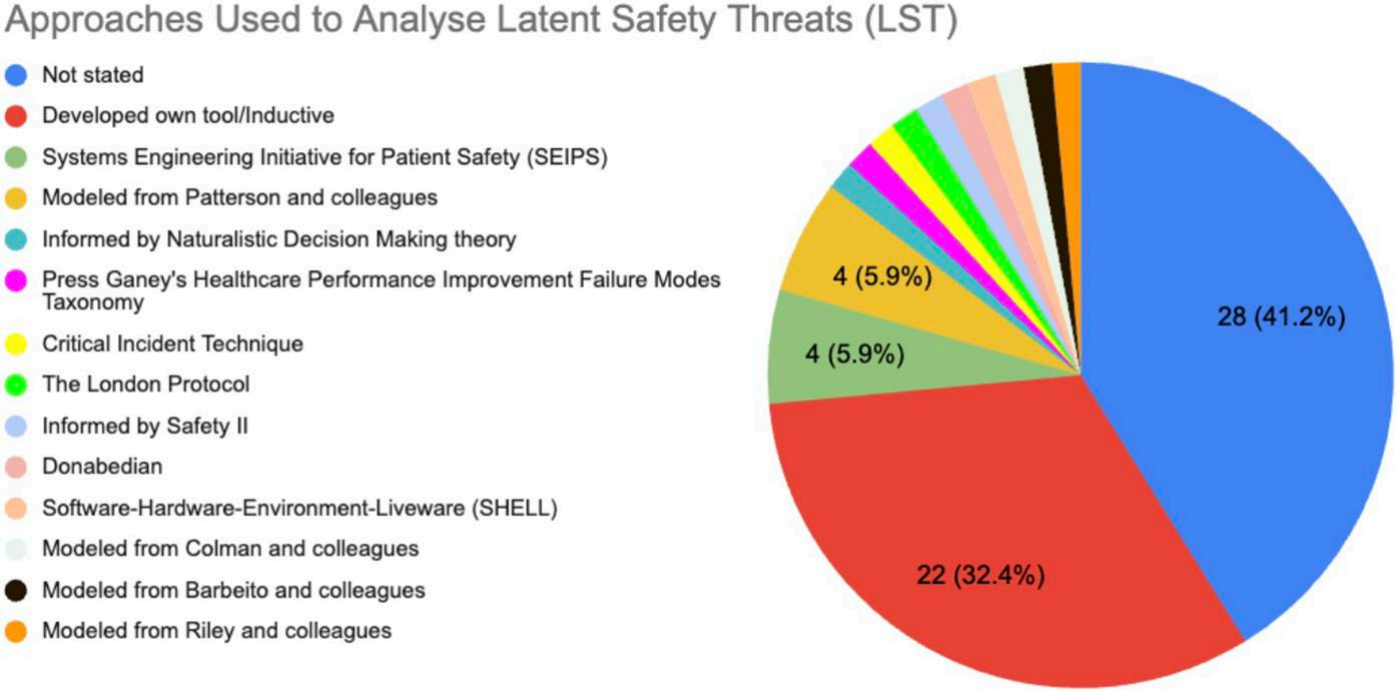
Approaches used to analyse latent safety threats (LSTs). Many did not state the approaches or frameworks used to analyse LSTs found (41.8%, 28/66) or developed their own approach (32.4%, 22/66).

41.8% (28/66) did not clearly state how they analysed LSTs. If there was not a clear distinction in the approaches used for identification vs. analysis, or authors did not clearly state that one approach was used for both identification and analysis, the approach was categorised according to how the current authors thought it was primarily used. Inductive development of a bespoke tool was the most common approach (33.3%, 22/66), followed by use of approaches from Patterson and colleagues (2) (6.1%, 4/66) and Systems Engineering Initiative for Patient Safety (SEIPS) (6.1%, 4/66) (2, 8). See Figure 3.

##### Visibility of system theory

The visibility of system theory was developed based on an inductive review of the included papers. Three categories were identified: Limited visibility, partial visibility, and full visibility. Limited visibility was defined as an absence of systems theory in the study. Partial visibility was defined as inconsistent or implicit use of systems theory. Full visibility was defined as the use of theory to influence the full study.

24 (36.3%) studies were categorised as “unclear/no visibility of systems theory”. 29 (43.9%) studies were categorised as “partial visibility of systems theory”. 13 (19.7%) studies were categorised as “full visibility of systems theory”.

##### Case studies: visibility of systems theory

Quantitative analysis revealed a broad spectrum of degrees of visibility of systems theory in the articles reviewed. The three case studies below expand on the quantitative categorisation to better understand how theory was used to inform research decisions and where opportunities for improvement exist. Case study 1 illustrates an example of unclear/no visibility of systems theory. Case study 2 illustrates an example of partial visibility of systems theory. Case study 3 illustrates an example of full visibility of systems theory (see Boxes 1–3).

Box 1 **An example of unclear/no visibility of systems theory to identify and analyse LSTs.**

Gable and colleagues aimed to improve team role clarity and identify LSTs in primary care (42). However, the degree of theoretical visibility was deemed “Unclear/no visibility”. This interpretation is based on what was reported and theory may have informed the research itself but was not conveyed in the final report.

The following questions to enhance theoretical visibility are based on this article by Gable and colleagues (42), and provide a roadmap of how similar articles in the “Unclear/no visibility” category may better articulate their theoretical underpinnings:

*Introduction*

- How did systems theory inform the rationale for this study?
- Why was a simulation-based approach selected?

*Methods*

- How were the methods informed by the rationale in the Introduction?
- Who identified the LSTs? Why this individual/group?
- What approaches were used to identify and analyse the LSTs? Why?
- If an inductive approach was used, why was this preferred?
- Were the causes behind the LSTs explored further? If so, how?
- How did systems theory inform how recommendations were developed?
- Which LSTs were prioritised for improvement?
- How were biases or assumptions acknowledged?

*Discussion*

- How was systems theory used to compare findings with similar work?
- Is this work generalisable to other settings?
- How did theory inform the limitations?

Box 2 **An example of partial visibility of systems theory to identify and analyse LSTs.**

Kaba and Barnes (2019) conducted a large-scale commissioning simulation to identify latent safety threats (LSTs) in a newly constructed 300-bed healthcare facility in Alberta, Canada (34). The degree of theoretical visibility was deemed “Partial visibility”. This interpretation is based on what was reported.

*Introduction*

- Human factors theory, specifically SEIPS, was explicitly included in the Introduction to emphasise the importance of these system factors in simulation.
- The use of specifically process oriented simulation and its benefits in the commissioning of new healthcare environments.
- Authors also explicitly mentioned the importance of often excluded disciplines, such as quality and architecture teams, when aiming to conduct a thorough systems analysis for new buildings.

*Methods*

- Simulations were designed with content experts in each hospital speciality that received specific simulation faculty training as part of the interventions.
- It is not stated whether simulation scenarios were written with specific systems theory applied.
- SEIPS and SEIPS 2.0 categories were used when discussing examples of latent safety threats, however no reference is made to how the theory was explicitly applied to the results.
- The debriefing tool, PEARLs, is also referenced as the sole debriefing tool used, however it is not discussed how this was utilised by simulation facilitators.

*Discussion*

- The results discussed and examples given were informed by the systems theory mentioned above, however there was no formal reporting of how this informed the processes and why it was beneficial.

*Overarchingly*

- Throughout the paper multiple references are made that demonstrate an understanding of some safety and debriefing theory. However it would have been beneficial to explore how these theories had been applied at different stages of the simulation design and implementation.

Box 3 **An example of full visibility of systems theory to identify and analyse LSTs.**

Petrosoniak and colleagues used video-based framework analysis to identify LSTs and determine the feasibility of regular simulation sessions (21). The degree of theoretical visibility was deemed “full visibility”. Again, this interpretation is based on what was reported. The following points highlight how theory was used throughout the article.

*Introduction*

- Included references to resilience by describing the need to understand how conditions develop.
- Used the above rationale to justify their use of video recorded simulations for the identification of LSTs, as this would enable multidisciplinary discussion and minimise recall bias.
- Described the value of human factors-based approaches to enable a more holistic understanding.

*Methods*

- Data from previous mortality meetings and reported adverse events informed the scenarios.
- Human Factors specialists were responsible for LST identification, following the rationale in the introduction.
- LSTs were coded inductively and deductively to ensure all were captured. Themes and subthemes were developed to extract further nuance.
- LSTs represented in the framework analysis were interpreted to be informed by systems thinking, with use of themes such as capacity demand misalignments, design, and situation awareness.

*Discussion*

- Articulated the value of applying framework analysis to allow researchers to review all LSTs in the same subtheme and examine how the same LST impacts different scenarios.
- Interpreted their relatively high number of LSTs identified in comparison to previous studies by describing the value of the video review method and inclusion of human factors experts.

*Overarchingly*

- The rationale set out in the introduction informed the methodological approaches. The Discussion section referenced the original aims, reflected on the results in the context of wider literature, and highlighted limitations inherent in the methodology.

##### Strategies to address LSTs

80.3% (53/66) of articles described strategies to address the LSTs identified. Strategies varied from one-off actions to more robust strategies such as accountability structures. Strategies to address LSTs identified were inductively coded with a resultant eight codes. These strategies, along with their definitions and the number of papers, are described in Table 3. See case study 4 for an illustration of the use of systems theory to address LSTs in healthcare.

**Table 3:** Strategies authors used to address latent safety threats (LST) when LSTs were identified.

Strategy	Definition	Number of papers in which strategy was used *Articles can be counted more than once
Escalation pathways	Includes channels to escalate LSTs to appropriate leaders or action item owners (e.g., use of the incident reporting system).	21 (15,19,22,23,25,30,31,33,36,37,42–52)
One-off actions per LST	Bespoke actions for each LST identified	17 (4,5,16,21,27,28,35,38,49,50,53–59)
Not stated/Minimal detail	NA	13 (11,14,17,24,34,39,60–65)
Stakeholder involvement	Includes partnerships with leaders, safety departments, and other clinical departments for awareness of the LSTs identified. Characterised by multidisciplinary engagement outside of the immediate clinical area to better understand and address the LST (e.g., with families, with human factors experts).	13 (18,20,21,26,32,33,36,43,44,66–69)
Current and future simulations	Includes improvements being integrated into or recommended for future simulations, use of future simulations to collect additional data about LSTs, or real time problem solving in the current simulation.	12 (13,18,26,37,47,52,53,66,67,70–72)
Prioritisation and intervention strategies	Includes the use of frameworks (e.g., PDSA cycles) to act on LSTs, distinguishing between actionable and non-actionable LSTs, or assigning a threat level to assist in prioritisation.	11 (22,23,26,29–31,46,51,67,69,73)
Accountability structures	Includes assessments of interventions once implemented, closed loop communication of actions taken, assigning actions based on who is best to complete them, action monitoring and follow up, or tracking of recurrence.	9 (23,25,33,35,37,43,73–75)
Communication of learning pathways	Includes coordinated dissemination plans or use of stories.	5 (4,12,25,45,74)

Approaches used to address LSTs were inductively coded and resulted in eight codes. Authors most commonly escalated LSTs identified to leadership, followed by “one off” actions to address LSTs detected.

##### Risk scoring LSTs

Some authors risk scored the LSTs without clearly stating how these risk scores were used to address the LSTs [hence the discrepancy between the articles that risk scored the LSTs (14) and the articles that applied “Prioritisation and intervention strategies” to address LSTs (11)]. Of the 14 articles that risk scored the LSTs identified, five (35.7%) used Failure Modes and Effects Analysis (FMEA) methodology (43), followed by Healthcare Failure Modes and Effects Analysis (HFMEA) (4/14, 28.6%) (44), inductive approaches (3/14, 21.4%), the Survey Analysis for Evaluating Risk (SAFER) matrix (1/14, 7.1%) (45), and adoption of the approach from Dadiz and colleagues (1/14, 7.1%) (38) (see Box 4).

Box 4 **An example of full visibility of systems theory to address LSTs.**

Seneviratne and colleagues aimed to identify, track, and reduce LSTs related to COVID airway management in the emergency department. The degree of theoretical visibility was deemed “Full visibility of systems theory”.

The following points highlight how theory was used in their approach to addressing the LSTs identified:

- Used the SAFER matrix to score LSTs identified and used these scores to assign priority,
- Developed cause-and-effect diagrams to associate each LST with potential solutions,
- Assessed the quality of the interventions in implementation and sustainment phases using the Donabedian model,
- Applied The PDSA Framework To Guide Iterative Improvements Based On Lsts Identified.

## Discussion

### Summary of evidence

The current review aimed to understand how systems theory has been used to identify LSTs in simulation. We found a variety of terms used instead of “latent safety threat”. A number of the papers reviewed did not explicitly state how they identified or analysed LSTs. 80.3% described how the LSTs uncovered were addressed. Several developed “one off” solutions for each LST without explaining how these solutions were developed. Of those that included risk scoring as an approach to address LSTs, various risk scoring tools were used. Finally, there was a broad spectrum of the degree of theoretical visibility. A case study of robust practice is included, as well as reflective questions for future researchers to consider to improve their own theoretical visibility.

### Strengths and limitations

All papers underwent initial screening and full text review by two researchers with 40.0% of the papers undergoing extraction by two researchers. Disagreements were discussed and adjustments were made as needed.

Several papers did not meet our inclusion criteria but may be valuable to consider for future research. For example, there were many papers in which LSTs were incidental findings, the aim of the simulation was to “test” how risky known LSTs actually were (rather than uncovering unknown LSTs), or in which the simulation was used to train other staff members to identify LSTs themselves.

### Comparison with existing literature

We support Grace and O’Malley's assertion that many of the “research” studies in this area take on a QI, rather than research, angle (6). Many studies in this review were interpreted to be QI (46). Any intention to report a QI project must be overtly stated, as QI aims to improve the local context using existing evidence and does not aim to produce new knowledge.

If the intention is to conduct research, theoretical transparency is essential. Lack of theoretical clarity can overlook assumptions or errors in logic (47), lead to a disconnect between intervention components (47), and reduce transferability (48). While theory may have been used in the research itself, it should be explicit in the written research output. The case studies illustrate key questions that should be addressed for robust theoretical transparency for future studies aiming to use simulation to address LSTs. Overarchingly, a response to “To what extent are the questions, approaches, and interpretations congruent with one another through a theoretical paradigm?” should be clearly articulated.

Several authors do not state how they identified or analysed LSTs. Without clear reporting of methodology, it becomes difficult to determine the extent to which LSTs identified were the result of robust approaches or the result of serendipitous observations. Many articles appeared to exemplify quantitative-dominant thinking in that authors seemed to aim to objectively confirm the presence of LSTs without acknowledging theoretical underpinnings or biases in “what they were seeing” (7). Therefore, there is a risk of oversimplifying complex healthcare systems, chalking up LSTs to isolated incidents of error (rather than exploring the complex interactions that influence safety) and developing solutions that are not likely to reliably address the problem.

How a problem is framed influences the approach to resolving it and the potential for learning (49, 50). Without the application of systems and Just Culture (51) approaches, LSTs are more likely to be attributed to individual non-compliance. This leads to solutions focussed on modifying individual behaviour. Therefore, the moral responsibility is to implement reliable, system-based solutions that address LSTs at their root, rather than relying on interventions directed at individuals.

Finally, the variety in terms used can complicate comparison of LSTs over time or between settings. The terminological precision problem is not new in the patient safety space. For example, there has been a longstanding advocacy for greater precision in the terms “adverse event” and “medication error” (52, 53). While organisations have published taxonomies, how this guidance is used in practice remains unclear (53–55). The terminological precision is likely to become even more pressing as simulation becomes an important component of safety work.

### Implications for policy, education, practice and research

Identifying one term to use may not be feasible, given that contexts may have different needs. However, academics should explicitly identify the terminology used, its definition, and why it was selected to help trace origins of terms and enable greater comparison. Publishers should reinforce this expectation upon receiving papers related to this topic. Similarly, publishers should assess the degree of theoretical visibility in papers submitted to peer-reviewed journals and highlight examples of good practice. Using probing questions, developing templates, and providing examples can steer the degree of theoretical visibility expected of future research. Future researchers should consider increased precision in their data extraction. Some tools [e.g., SEIPS (8)] are more directly aimed at identifying LSTs whereas others [e.g., PEARLS (41)] provide the conditions for groups to discuss the LSTs but were not designed specifically to detect LSTs. Defining which tools are most appropriate based on the aim may better refine the findings.

Organisations should integrate simulation into the larger safety learning strategy. Historically, safety learning has been characterised by incident reports and investigations once a harm event has occurred. Techniques that provide additional insight into LSTs, such as simulation, could be further integrated into traditional safety strategies by encouraging reporting of LSTs identified in simulation in the normal event report systems or allocating safety resources to facilitate simulation debriefs, for example. Many of the papers reviewed “stumbled upon” LSTs, which is a testament to the benefit of systems testing via simulation. Simulation leaders should be aware that LSTs may be unexpectedly detected and should have an established process to escalate these LSTs to groups that will be able to help. A significant challenge arises from the fact that simulation often resides within the “education” division of healthcare organisations, rather than also being integrated into the “safety” division. This division can create barriers to leveraging simulation for system-level improvements. Bridging this gap might be achieved by aligning simulation efforts with organisational priorities and designing simulations for both education and systems improvement.

It is important to make recommendations to mitigate LSTs identified but further resources must be allocated to refine the solution once the LST is identified (56). Organisations conducting simulations should embed clear guidance to help translate LSTs into solutions (56). Those with safety expertise should be involved in simulation design, data collection and analysis, and recommendation generation. These professionals can serve as the “glue” between the simulation and larger organisational work.

Many articles involved high-fidelity simulations, which are costly and may disproportionately report on findings from high-income countries. There is a gap in the literature around how lower fidelity simulations can be used to identify LSTs in low- and middle-income countries (LMIC). However, certain costly aspects of the simulation, such as conceptualisation, do not need to be created in silos. Making scenarios, written materials, and data collection tools publicly available so that they can be adapted in other contexts could minimise the barriers to engagement for those in LMIC.

## Conclusion

This review has revealed the extensive use of simulation to aid in the identification of LSTs. Simulation practitioners should aim to make explicit their use of systems theory when designing simulations to identify LSTs and be prepared for the likelihood of identifying LSTs incidentally. Researchers should overtly state how theory informed their study. Theoretical transparency is likely to enhance the transferability of approaches and findings to other contexts, which is of particular value in settings where resources for simulation are scarce.

## Data Availability

The original contributions presented in the study are included in the article/Supplementary Material, further inquiries can be directed to the corresponding author.
