# Supplementary material for: The use of healthcare simulation to identify and address latent safety threats: a scoping review
Source: Front Health Serv. 2025 Nov 18;5:1682629. doi: 10.3389/frhs.2025.1682629 (PMC12669178; doi:10.3389/frhs.2025.1682629)
Supplement: Supplementary file 1 [file Supplementaryfile1.docx]

**Medline (Ovid MEDLINE® Epub Ahead of Print, In-Process & Other Non-Indexed Citations, Ovid MEDLINE® Daily and Ovid MEDLINE®) 1946 to present**

1 exp Risk Assessment/ 313734

2 (latent adj1 (threat* or hazard* or safety or error* or risk* or event*)).ti,ab,kf. 547

3 (system adj1 (threat* or hazard* or safety or error* or risk* or event*)).ti,ab,kf. 2933

4 (safety adj1 (threat* or hazard* or error* or risk* or event*)).ti,ab,kf. 8196

5 1 or 2 or 3 or 4 324404

6 risk*.ti,ab,kf. 3038248

7 safety.ti,ab,kf. 731244

8 1 or 6 or 7 3709367

9 exp Simulation Training/ 11900

10 simulat*.ti,ab,kf. 728892

11 (learn* or teach* or educat* or train* or coach*).ti,ab,kf. 2012236

12 exp Education/ 911664

13 11 or 12 2472610

14 10 and 13 75821

15 9 or 10 731161

16 Manikins/ 5718

17 manikin*.ti,ab,kf. 3883

18 mannequin*.ti,ab,kf. 2337

19 task based.ti,ab,kf. 3655

20 Role Playing/ 2306

21 role play*.ti,ab,kf. 24852

22 walk through*.ti,ab,kf. 733

23 tabletop.ti,ab,kw. 1203

24 16 or 17 or 18 or 19 or 20 or 21 or 22 or 23 40766

25 13 and 24 12095

26 virtual reality/ 6120

27 virtual realit*.ti,ab,kw. 18763

28 26 or 27 19759

29 13 and 28 9306

30 9 or 14 or 25 or 29 90605

31 5 and 30 799

**Embase 1974 to present (EXP ONLY, NOT FOCUSED)**

Embase 1974 to present

1 exp risk assessment/ 759790

2 (latent adj1 (threat* or hazard* or safety or error* or risk* or event*)).ti,ab,kf. 808

3 (system adj1 (threat* or hazard* or safety or error* or risk* or event*)).ti,ab,kf. 4080

4 (safety adj1 (threat* or hazard* or error* or risk* or event*)).ti,ab,kf. 11747

5 1 or 2 or 3 or 4 773801

6 risk*.ti,ab,kf. 4392041

7 safety.ti,ab,kf. 1119845

8 1 or 6 or 7 5394210

9 exp simulation training/ 10184

10 simulat*.ti,ab,kf. 776753

11 (learn* or teach* or educat* or train* or coach*).ti,ab,kf. 2589744

12 exp education/ 1706010

13 11 or 12 3399012

14 10 and 13 97740

15 9 or 10 778583

16 exp manikin/ 3628

17 manikin*.ti,ab,kf. 5518

18 mannequin*.ti,ab,kf. 3556

19 task based.ti,ab,kf. 4584

20 exp role playing/ 17584

21 role play*.ti,ab,kf. 29593

22 walk through*.ti,ab,kf. 951

23 tabletop.ti,ab,kw. 1328

24 16 or 17 or 18 or 19 or 20 or 21 or 22 or 23 60272

25 13 and 24 19092

26 exp virtual reality/ 28091

27 virtual realit*.ti,ab,kw. 23216

28 26 or 27 34397

29 13 and 28 15193

30 9 or 14 or 25 or 29 122167

31 5 and 30 2146
